# The chromosome-scale reference genome for the pinfish (*Lagodon rhomboides*) provides insights into their evolutionary and demographic history

**DOI:** 10.1093/g3journal/jkae096

**Published:** 2024-05-13

**Authors:** Katherine M Eaton, Trevor J Krabbenhoft, Nathan J C Backenstose, Moisés A Bernal

**Affiliations:** Department of Biological Sciences, Auburn University, Auburn, AL 36849, USA; Department of Biological Sciences, University at Buffalo, Buffalo, NY 14260, USA; Research and Education in Energy, Environment, and Water (RENEW) Institute, University at Buffalo, Buffalo, NY 14260, USA; Department of Biological Sciences, University at Buffalo, Buffalo, NY 14260, USA; Department of Biological Sciences, Auburn University, Auburn, AL 36849, USA; Smithsonian Tropical Research Institute (STRI), Panama City, 0843-03092, Panama

**Keywords:** genomics, marine biology, diet, life history, nanopore sequencing, climate change

## Abstract

The pinfish (*Lagodon rhomboides*) is an ecologically, economically, and culturally relevant member of the family Sparidae, playing crucial roles in the marine food webs of the western Atlantic Ocean and Gulf of Mexico. Despite their high abundance and ecological importance, there is a scarcity of genomic resources for this species. We assembled and annotated a chromosome-scale genome for the pinfish, resulting in a highly contiguous 785 Mb assembly of 24 scaffolded chromosomes. The high-quality assembly contains 98.9% complete BUSCOs and shows strong synteny to other chromosome-scale genomes of fish in the family Sparidae, with a limited number of large-scale genomic rearrangements. Leveraging this new genomic resource, we found evidence of significant expansions of dietary gene families over the evolutionary history of the pinfish, which may be associated with an ontogenetic shift from carnivory to herbivory seen in this species. Estimates of historical patterns of population demography using this new reference genome identified several periods of population growth and contraction which were associated with ancient climatic shifts and sea level changes. This genome serves as a valuable reference for future studies of population genomics and differentiation and provides a much-needed genomic resource for this western Atlantic sparid.

## Introduction

With the advent of long-read sequencing, biologists can now generate complete genome sequences for virtually any organism of interest. Yet despite the relative ease and speed with which we are now able to sequence full genomes, certain taxonomic groups remain underrepresented in terms of genomic resources (David *et al*. 2019; Hotaling *et al*. 2021). Notably, although ray-finned fishes (Class: Actinopterygii) comprise over 50% of extant vertebrate biodiversity, only 3.4% of species have publicly available genomic resources (May 2023; NCBI Genome, www.ncbi.nlm.nih.gov/genome). Even among the small fraction of sequenced Actinopterygians, biases remain, with many sequenced species being those of major commercial interest, either for the seafood industry or the aquarium trade. Given the advancements in sequencing technology and computational power seen in the past several decades, it is crucial to refocus efforts toward investigating the genomes of fish species that are of both commercial and ecological relevance.

Seabreams and porgies (Family: Sparidae) are commonly recognized as valuable food fishes, particularly in Europe and Asia (Jobling and Peruzzi 2010; Basurco *et al*. 2011). Given their commercial relevance, previous research has focused on generating genomic resources for farmed and cultured sparids, including the gilthead seabream in the Mediterranean (*Sparus aurata*; Pauletto *et al*. 2018) and the red seabream (*Pagrus major*; Shin *et al*. 2018), Chinese black porgy (*Acanthopagrus schlegelii*; Z. Zhang *et al.* 2018), and yellowfin seabream (*Acanthopagrus latus*; Zhu *et al*. 2021) in East Asia. Comparatively little genomic research has focused on western Atlantic sparids, presumably due to being lesser interest in farming or consumption of seabreams and porgies in the Americas. Yet while sparids of the western Atlantic may be less desirable for aquaculture and consumption, there are several species of commercial and ecological importance. One such species, the pinfish (*Lagodon rhomboides*), plays a key role in ecosystems of the Gulf of Mexico and western Atlantic while also being both economically and culturally important to local communities.

**Fig. 1.**
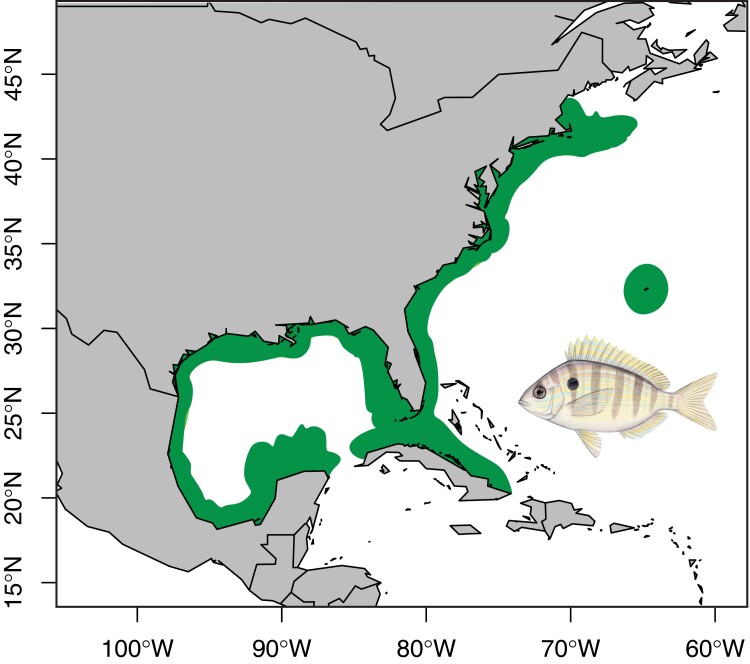
Approximate geographic distribution of the pinfish (green). Distribution map based on geographic range information from the International Union for the Conservation of Nature (IUCN) (Russell *et al*. 2014). Inset: Drawing of the pinfish by Julie Johnson, Life Sciences Illustrations.

Pinfish are broadly distributed in coastal ecosystems of the Gulf of Mexico and western Atlantic Ocean (Fig. 1, Russell *et al*. 2014), and are by far the most abundant species of fish found in seagrass beds of the Gulf of Mexico during the summer months (Stallings and Koenig 2011). Due to their abundance, pinfish are commonly used as bait for local fishers targeting large commercial fishes, such as red drum, tarpon, snook, and grouper (Darcy 1985). Beyond their utility as a bait species, these fish also provide ecosystem services to both the nearshore and offshore areas that they inhabit, serving as a key member of the food web, due to their high abundance and trophic position. Larval and juvenile pinfish develop in shallow, nearshore seagrass beds during the first 8–9 months of their lives, where they are exclusively carnivorous, consuming copepods, amphipods, shrimp, mysids, and other invertebrates (Stoner 1980; Darcy 1985). As they develop into adults, they undergo an ontogenetic shift in diet, transitioning to a primarily herbivorous lifestyle (Darcy 1985; Gallagher *et al*. 2001; Barbosa and Taylor 2020). While ontogenetic shifts in diet are common among fishes, they are frequently associated with increases in body size and the capacity to consume larger prey items (e.g. planktivorous larvae transition to consuming macroinvertebrates or fishes as juveniles and adults; Sánchez-Hernández *et al*. 2019). Ontogenetic transitions from carnivory to herbivory, as observed in the pinfish, appear to be relatively unusual, which warrants more in-depth research. Also during development, juvenile pinfish shift their preferred habitat, migrating to cooler, deeper offshore areas where they will eventually spawn (Caldwell 1957; Darcy 1985). This migration facilitates the export of biomass accumulated from inshore primary producers to oligotrophic, nutrient-limited offshore systems (Nelson *et al*. 2013). As pinfish are preyed upon by large piscivorous fish, seabirds, and marine mammals (Darcy 1985), they increase nutrient availability in these offshore ecosystems, allowing them to sustain a higher biomass (Nelson *et al*. 2013). In some areas of the Gulf of Mexico, pinfish alone have been estimated to comprise ∼25% of the total nitrogen content available to higher trophic levels in offshore food webs (Nelson *et al*. 2013), further highlighting that this species represents a major source of energy for oligotrophic offshore systems. Despite the biological relevance of the pinfish, there is a paucity of genomic resources available, which hinders our ability to both understand the evolutionary history and predict the future persistence of this species.

Previous work suggests that juvenile pinfish are sensitive to marine heatwaves, showing physiological and molecular responses to acute heat stress (Eaton *et al*. 2022). As anthropogenic climate change is increasing the frequency and intensity of heat stressors such as oceanic heatwaves (Frölicher *et al*. 2018; Oliver *et al*. 2018), the potential for persistence of this species remains to be fully evaluated. It is imperative to develop genomic resources to further advance our understanding of the population dynamics of this species, and how they may continue to be affected by changes in ocean temperature. With this in mind, here, we present the first chromosome-scale genome assembly and annotation for the pinfish. These new genomic resources provide insights into the dynamic evolutionary and demographic history of the species, and they allow us to propose links between genomic changes and the evolution of unique phenotypes. Furthermore, this study provides a new genomic resource for coastal fishes of the western Atlantic, which can be leveraged for future studies examining patterns of local adaptation, population connectivity, and the potential for persistence of this keystone species in the face of a rapidly changing world.

## Methods

### Sample collection

Pinfish for whole genome sequencing were collected by seine and cast net from Walker Island, Perdido Bay, Alabama (30.384N, 88.314W) in February and September of 2020. Individuals were euthanized via cervical dislocation and dissected. Gill, liver, and muscle tissues from dissected fish were preserved in 95% ethanol or DNA/RNAShield (Zymo) and stored at −20°C. In November 2020, an additional pinfish was collected from Dauphin Island, Alabama (30.251N, 88.080W), euthanized by cervical dislocation, and frozen whole for Hi-C scaffolding. All collections were done with the permission of the Alabama Department of Natural Resources (Permit 2021-3-01) and the Institutional Animal Care and Use Committee of Auburn University (Protocol 2020-3708).

### DNA extraction and sequencing

For short-read sequencing, genomic DNA was extracted from the gills and liver of a single adult pinfish. DNA extractions were carried out using the Invitrogen PureLink Genomic DNA Mini Kit, following the manufacturer's protocol. DNA was quantified using an Invitrogen Qubit 4 Fluorometer, and quality was visually checked on a 1% agarose gel. High-quality DNA was sent for library preparation and 150-bp paired-end Illumina sequencing at Novogene Corporation (Sacramento, CA, USA). For nanopore sequencing, high molecular weight genomic DNA was extracted from the gills and muscles of a second pinfish. High molecular weight DNA was extracted using the Qiagen Genomic-tip 500/G kit, following the manufacturer's protocol, with slight modifications (Supplemental Methods). DNA was quality-checked visually on a 1% agarose gel and quantified using an Invitrogen Qubit 4 Fluorometer. High molecular weight DNA was size-selected for fragments larger than 10 kb using a Circulomics Short Read Eliminator Kit. DNA libraries were prepared with both raw and size-selected DNA using the Oxford Nanopore SQK-LSK109 ligation sequencing kit and run on 4 MinION flow cells (sequenced on a GridION machine). Nanopore base calling was completed using the high-accuracy base calling model in Guppy Basecalling Software (Oxford Nanopore Technologies) v. 4.0.11, called from MinKNOW v. 20.06.9 and v. 20.06.17.

### Assembly

Raw nanopore reads were assembled using Flye v. 2.8-b1674 (Kolmogorov *et al*. 2019). Illumina reads were trimmed to remove adapters and low-quality sequences using the program cutadapt v 2.3 (Martin 2011) and were then used to polish the initial assembly. Briefly, reads were mapped to the preliminary assembly using the program BWA-mem v. 0.7.12-r1039 (Li 2013). Output sequence alignment map (SAM) files were converted to binary alignment map (BAM) format, sorted, and indexed using samtools v. 1.9 (Li *et al*. 2009). Pilon v. 1.23 (Walker *et al*. 2014) was then used to polish the assembly, using the BAM alignments. Full details of the assembly parameters used can be found in the Supplemental Methods.

The draft genome assembly was sent to Phase Genomics (Seattle, Washington DC, USA), along with the frozen tissue of a third-adult pinfish, for Proximo Hi-C scaffolding, to produce a chromosome-scale assembly. To remove any unscaffolded contigs that were the result of microbial or nontarget reads, the program Kraken 2 v. 2.1.2 (Wood *et al*. 2019) was used to create a custom taxonomic sequence classification database and assess the origin of all contigs in the pinfish genome. Contigs classified as nonfish in origin (presumably from microbial contaminants) were removed from the assembly using the program SeqKit v. 0.14.0 (Shen *et al*. 2016). The resulting scaffolds were ordered according to length and renamed. The final assembly was assessed for completeness using BUSCO v. 5.1.2 (Manni *et al*. 2021), searching against the *actinopterygii_odb10* database, using the gene predictor MetaEuk (Levy Karin *et al*. 2020).

### Annotation

We used RepeatModeler v. 2.0.1 (Flynn *et al*. 2020) to characterize transposable element families in our genome. Repeat families identified from RepeatModeler were used as a custom repeat library for repeat masking by RepeatMasker v. 4.1.1 (Smit *et al*. 2013–2015). We then performed 2 rounds of genome annotation using MAKER v. 3.01.03 (Cantarel *et al*. 2008; Supplemental Methods), supplying repeat evidence from RepeatModeler, protein evidence from the gilthead seabream (*S. aurata*) and the zebrafish (*Danio rerio*) obtained from Ensembl (Howe *et al*. 2021, www.ensembl.org, Ensembl release 103), and expressed sequence tag evidence using the transcriptome assembly for *L. rhomboides* (Eaton *et al*. 2022). To validate our results, we used InterProScan v. 5.52–86.0 (Jones *et al*. 2014) to identify proteins in our annotation that contained at least one recognizable protein domain found in the Pfam database of protein families.

Predicted proteins from the MAKER output were annotated in a series of iterative BLAST searches for sequence similarity to proteins from publicly available fish genomes. Using the program BLAST + v. 2.13.0 (Altschul *et al*. 1990; Camacho *et al*. 2008), we searched the predicted proteins from the *L. rhomboides* genome against a database of peptide sequences from the *S. aurata* genome downloaded from Ensembl (Cunningham *et al*. 2022, Ensembl release 107), using the command “blastp” and specifying an *e*-value cutoff of 1*e*^−10^. BLAST matches were filtered to only include high-quality matches (i.e. matches with query coverage > 50% and percent identity ≥ 75%) and the best match for each protein was identified based on the highest bit-score. This process was then repeated for predicted proteins in the pinfish genome that did not have a high-quality match in the *S. aurata* database, using the same search parameters against peptides from the large yellow croaker genome (*Larimichthys crocea*) and then the zebrafish genome (*Danio rerio*), downloaded from Ensembl (Cunningham *et al*. 2022, Ensembl release 107).

### Synteny

To examine genomic structure and large-scale chromosomal rearrangements over evolutionary time, we used the program SynMap2 (Haug-Baltzell *et al*. 2017) with default parameters implemented in CoGe (Lyons and Freeling 2008) on coding sequences, comparing the structure of the pinfish genome to that of the gilthead seabream (*S. aurata*; NCBI assembly accession number: GCA_900880675.1), the yellowfin seabream (*A. latus*; NCBI assembly accession number: GCF_904848185.1), and the Japanese puffer (*Takifugu rubripes*; NCBI assembly accession number: GCF:_901000725.2). Syntenic matches were plotted using Circos v. 0.69–9 (Krzywinski *et al*. 2009). To aid in visualization and interpretation of the synteny results, we also generated synteny plots using the program D-GENIES (Cabanettes and Klopp 2018), which generates dot plots of genomic alignments created via the program Minimap v. 2.26 (Li 2018). Large inversions (>4 Mb in length) were identified on the resulting plots and then filtered to exclude those which may be the results of scaffolding errors. Briefly, we determined the genomic coordinates of the putative inversion breakpoints and compared them with our scaffolding data. For those instances where one or more breakpoints coincided with a gap between scaffolded contigs, we assumed the possibility of a scaffolding error and removed that inversion from downstream analyses. This approach, while conservative, minimizes the possibility of a Type I error in inversion detection. We then used Fisher's exact test to determine if certain Gene Ontology (GO) terms were overrepresented among the set of genes found in each inversion, as compared to the rest of the genome.

### Gene family evolution

To contextualize the functional genomics and evolutionary history of the pinfish, we assessed phylogenetic patterns of gene family expansion and contraction using CAFE v. 5.0 (Mendes *et al*. 2020). This program uses a birth/death process to model gains and losses of genes in particular gene families across a time-calibrated phylogenetic tree, inferring the most likely number of genes in each gene family at every internal node of the phylogeny. It then estimates a maximum-likelihood global evolutionary rate for all gene families across the tree. Then, for each gene family, every branch of the phylogeny is assessed to determine if the rate of gene gain or loss is significantly greater than the global rate, which indicates a significantly rapid gene family expansion or contraction at that point in time. We downloaded peptide sequences from 18 publicly available teleost fish genomes (Supplementary Table 1) and isolated the longest isoform of each gene in each species using the python script “longest_iso.py” provided by the CAFE developers, in addition to SeqKit v. 0.14.0 (Shen *et al*. 2016). We ran the program OrthoFinder v. 2.4.0 (Emms and Kelly 2019) with default parameters to identify families of orthologous genes and build a phylogeny for use in CAFE. The output table containing the gene counts per orthogroup for each species was filtered to remove gene families with large copy number variance (where one or more species had 100 or more copies of a particular gene, as recommended by the CAFE developers), and then the filtered table was used as input for CAFE.

To generate a time-calibrated phylogenetic tree for use in CAFE, we first identified single-copy orthologues that were present in all 19 species, using the output of OrthoFinder. We aligned the coding sequences for each single-copy orthogroup using the program MACSE v. 2.06 (Ranwez *et al*. 2018) and constructed a maximum-likelihood phylogenetic tree using IQ-TREE v. 1.6.12 using ModelFinder for extended model selection (Nguyen *et al*. 2015; Kalyaanamoorthy *et al*. 2017; Hoang *et al*. 2018). The gene trees for each orthogroup were used to infer the species tree using ASTRAL v. 5.7.8 (C. Zhang et al. 2018). We generated a final maximum-likelihood tree from a concatenated alignment of all single-copy orthologues also using IQ-TREE, partitioning by locus and using the previously identified best-fit substitution model for each orthologue (Chernomor *et al*. 2016) and constraining the topology of the tree to match that of the ASTRAL-generated species tree. This final maximum-likelihood tree was time-calibrated using the program treePL (Smith and O’Meara 2012), using seven fossil calibrations (Supplementary Table 2).

We ran CAFE v. 5.0 (Mendes *et al*. 2020), using the table of gene counts per orthogroup (or gene family) as identified by OrthoFinder and the ultrametric phylogeny, to identify gene families that have undergone significantly rapid expansions or contractions in gene copy number over evolutionary time. An initial run of CAFE estimated a model accounting for genome assembly error across the 19 taxa (specifying the parameter -*e*). This error model was then used in a second round of CAFE, to determine the global gene birth–death rate (*λ*) with two discrete evolutionary rate categories (-*k* 2). The output from CAFE was filtered manually to identify gene families that were significantly rapidly expanding or contracting (*P* < 0.05). To further investigate the functional significance of these families, we identified GO categories that were overrepresented among the significantly rapidly evolving gene families using Fisher's exact test, based on the bioinformatic pipeline and code provided by Wright *et al*. (2015).

### Demographic history

To examine patterns of historical population demography in the pinfish, we used the Pairwise Sequentially Markovian Coalescent (PSMC) model (Li and Durbin 2011). PSMC estimates historical recombination events and the most recent common ancestor (MRCA) of alleles at a given locus across a single diploid genome to infer the coalescent rate within a time period, which has an inverse relationship to effective population size (*N_e_*, Li and Durbin 2011). Briefly, we mapped the trimmed and cleaned Illumina reads back to the final genome assembly using BWA-mem v. 0.7.17-r1188 (Li 2013). Aligned reads were sorted and indexed using samtools v. 1.7 (Li *et al*. 2009). We called SNPs for this alignment using bcftools v. 1.7 (Li 2011). The resulting variant call format (VCF) file was converted into FASTQ format using the command “vcfutils.pl vcf2fq” from bcftools (Li 2011). This FASTQ file was used in PSMC v. 0.6.5-r67 (Li and Durbin 2011), estimating historical effective population size. We first did an initial run, specifying -N25 (setting the maximum number of iterations to 25), -t5 (setting the maximum 2N0 coalescent time to 5), -r5 (setting the initial *θ*/*ρ* ratio to 5), and -*P* “1*4 + 25*2 + 1*4 + 1*6” (setting the atomic time intervals), based on parameters previously applied to similar analyses in three spine stickleback (Liu *et al*. 2016; Kirch *et al*. 2021). We then performed 100 bootstrap replicates, using the same parameters as above. The results were scaled to real time and plotted, assuming a generation time of 1.38 years, based on von Bertalanffy growth parameters for *L. rhomboides* estimated by Nelson (2002), and a mutation rate of 3.7*10^−8^ substitutions/nucleotide/generation (Kirch *et al*. 2021).

Full details about the bioinformatic commands and parameters used during assembly, annotation, and subsequent analyses are available in the Supplemental Methods. The scripts used to assemble the genome are available online at: https://github.com/kmeaton/Pinfish_Genome.

## Results

### Assembly

Illumina sequencing resulted in 260,787,246 read pairs comprising 78.1 Gb of sequence data after read trimming and quality filtering. Nanopore sequencing resulted in an additional 9,281,835 reads (40.9 Gb). Raw reads from both the Illumina and Nanopore sequencing are deposited in NCBI's GenBank under BioProject accession number PRJNA1039866. The initial long-read assembly generated by Flye and polished using Pilon was 795.8 Mb in length consisting of 4,531 contigs with a contig N50 of 3.53 Mb. The final (haploid) assembly is comprised of 3,522 contigs, 840 of which were scaffolded into 24 chromosomes (Table 1). Approximately 754.7 Mb of the 785.3 Mb total assembly length (96.10%) is contained in the 24 scaffolded chromosomes (scaffold N50: 31.4 Mb, Table 1). The final assembly (Lrho_1.0) is also deposited in GenBank under accession number JBBNBP000000000. BUSCO results indicate high completeness; out of the 3,640 single-copy orthologues in the *actinopterygii_odb10* database, 3,540 (97.3%) were identified as complete and single-copy, 58 (1.6%) were complete and duplicated, 9 (0.2%) were fragmented, and 33 (0.9%) were missing in our genome (Table 1).

**Table 1.** Assembly and summary statistics for the pinfish genome and other publicly available genomes for species in family Sparidae.

Species	Assembly length (Mb)	Number of contigs	Contig N50 (Mb)	Scaffold N50 (Mb)	BUSCO % complete	Reference
*Lagodon rhomboides*	785.3	3,522	3.53	31.4	98.9	This study
*Acanthopagrus latus*	685.1	214	14.9	30.7	99.6	NCBI accession: GCF_904848185.1
*Sparus aurata*	833.6	1,223	2.9	35.8	99.3	NCBI accession: GCF_900880675.1
*Acanthopagrus schlegelii*	688.1	115,091	0.017	7.6	89.1	Z. Zhang *et al*. 2018
*Diplodus sargus*	785	2,408,078	1.10	3.37	96.6	Fietz *et al*. 2020
*Pagrus major*	829.3	1,657	2.8	N/A	97.1	Shin *et al*. 2018

### Annotation

RepeatMasker identified 235.3 Mb of repetitive content across the pinfish genome (29.97% of total genome length; Table 2). In total, 24,634 protein-coding genes were identified using MAKER, with an average gene length of 11,840 bp. Of these 24,634 genes, 20,390 (82.77%) contained at least one protein domain found in the Pfam database. Additionally, 19,703 of these protein-coding genes (79.98%) were successfully annotated based on sequence similarity to known proteins from the *S. aurata*, *L. crocea*, and *D. rerio* genomes.

**Table 2.** Repetitive element content of the *Lagodon rhomboides* genome.

Repeat type	Number detected	Length of genome occupied (Mb)	Percentage of genome occupied (%)
SINEs	8,861	1.07	0.14
LINEs	62,800	18.21	2.32
LTR elements	19,870	8.39	1.07
DNA transposons	328,494	64.15	8.17
Unclassified	635,485	121.22	15.43

### Synteny

At a broad phylogenetic scale, we saw strong patterns of conserved macrosynteny between the pinfish and the Japanese puffer (*T. rubripes*, Supplementary Fig. 1). While intrachromosomal rearrangements were common, there was minimal evidence of translocations between chromosomes. Interestingly, we see 2 putative instances of chromosomal fission or fusion events (chromosomes 7 and 21 in *L. rhomboides* correspond to chromosome 1 in *T. rubripes*, and chromosomes 23 and 24 in *L. rhomboides* correspond to chromosome 8 in *T. rubripes*; Supplementary Fig. 1).

**Fig. 2.**
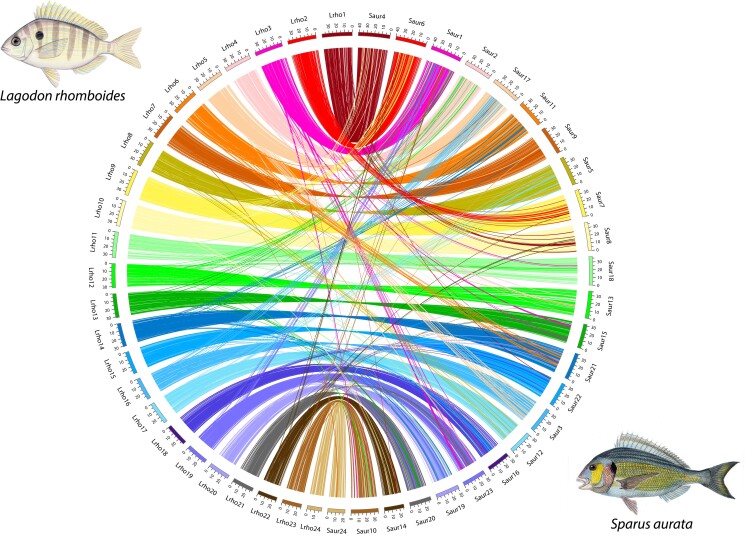
Circos plot showing syntenic regions between the genome of the pinfish (left) and *S. aurata*, the gilthead seabream (right). Links between blocks of the same color indicate conserved gene order on homologous chromosomes, while links between blocks of different colors indicate genomic translocations between nonhomologous chromosomes. *S. aurata* image reproduced under Creative Commons License by Werner—Histoire Naturelle des Poissons, Public Domain.

Within the family Sparidae, synteny was strongly conserved between the pinfish and *S. aurata* (Figs. 2; Supplementary Fig. 2). Across the 24 chromosomes there was generally strong collinearity, however, we identified 2 large-scale (>4 Mb) inversions between the 2 genomes (Supplementary Table 3, Supplementary Figs. 2 and 3). We found similar results between the pinfish and *A. latus*, with high collinearity across chromosomes, and two large-scale (>4 Mb) inversions (Supplementary Table 4, Supplementary Figs. 4 and 5). The two inversions identified between the pinfish and *A. latus* genomes overlapped virtually completely with the two large-scale inversions seen in the pinfish vs *S. aurata* comparison (Supplementary Tables 3 and 4, Supplementary Figs. 2–5).

For each of these two major inversions, a Fisher's exact test was used to assess if genes with certain GO terms were significantly enriched within the inversion when compared to the remainder of the genome. While the inversion on *L. rhomboides* chromosome 23 was not significantly enriched for any GO terms, the 4.1 Mb inversion near the center of *L. rhomboides* chromosome 13 was significantly enriched for several GO terms associated with nervous and sensory system function and development, including “auditory receptor cell development”, “detection of mechanical stimulus”, and “neural crest cell migration” (Supplementary Table 5).

### Gene family evolution

**Fig. 3.**
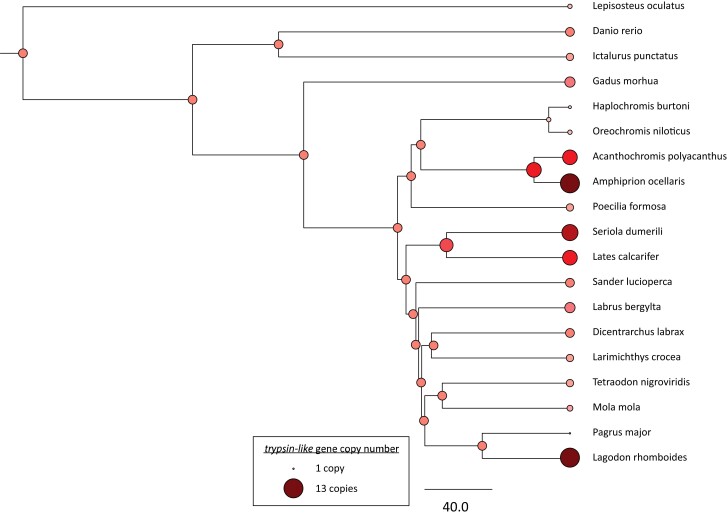
Evolution of *trypsin-like* gene family across the fish tree of life. Colored circles at each node and tip of the phylogeny indicate the estimated number of gene copies in the gene family at that node/tip. Smaller, lighter colored circles are indicative of fewer gene copies, and larger, darker colored circles are indicative of more gene copies. Phylogeny is scaled in millions of years, with the scale bar representing 40.0 million years.

We identified 1,981 single-copy orthologues that were present in all 19 species used for our gene family evolution analyses and used the coding sequences for these single-copy orthologues to generate a time-calibrated phylogenetic tree (Fig. 3). Using this phylogenetic tree, we estimated rates of gene family evolution for 14,619 gene families. The average global rate of gene birth/death (*λ*) across the tree was estimated as 0.00144 gains/losses per million years. Out of the 14,619 gene families examined, 503 were determined to be significantly rapidly contracting or expanding (*P* < 0.05) for at least 1 branch within the phylogeny.

Along the terminal branch of *L. rhomboides*, we detected 112 gene families with significantly rapid changes in gene copy number, 81 of which were expanding and 31 of which were contracting (*P* < 0.05). Among the most highly expanded gene families unique to the pinfish, we saw families associated with dietary function, such as *lactase-phlorizin hydrolase* (two copies in the MRCA of *L. rhomboides* and *P. major* expanded to 5 copies in *L. rhomboides*) and *trypsin-like* (6 copies in the MRCA of *L. rhomboides* and *P. major* expanded to 13 copies in *L. rhomboides*, Fig. 3). Interestingly, 11 of the 13 *tryspin-like* genes identified in the *L. rhomboides* genome are clustered within a 57-kb window of chromosome 5. Additionally, gene families which were significantly rapidly evolving (i.e. expanding or contracting) in the pinfish were enriched for GO terms associated with immune system function, such as “immune response” “complement activation,” and “immune effector process,” as well as GO terms associated with chromatin structure, such as “nucleosome organization”, “chromosome”, and “DNA packaging complex”.

In the branch leading to family Sparidae, we identified 76 rapidly evolving gene families, 42 of which were significantly expanded, and 34 of which were significantly contracted (*P* < 0.05). Eight of these gene families were olfactory receptor families (6 contracted, 2 expanded; *P* < 0.05). GO term analysis also indicated that gene families involved in sensory perception and olfaction were significantly rapidly changing in copy number along this branch of the tree, with rapidly evolving families being significantly enriched for terms such as “sensory perception of smell” and “olfactory receptor activity”. Other rapidly expanding or contracting gene families in the branch leading to family Sparidae include digestive enzyme families containing genes such as *trypsin-3* and *duodenase-1* (expanded from three copies in the MRCA of Tetraodontiformes and Sparidae to 7 copies in the MRCA of family Sparidae), and *pepsin-A* (expanded from 2 copies in the MRCA of Tetraodontiformes and Sparidae to 3 copies in the MRCA of family Sparidae).

### Demographic history

**Fig. 4.**
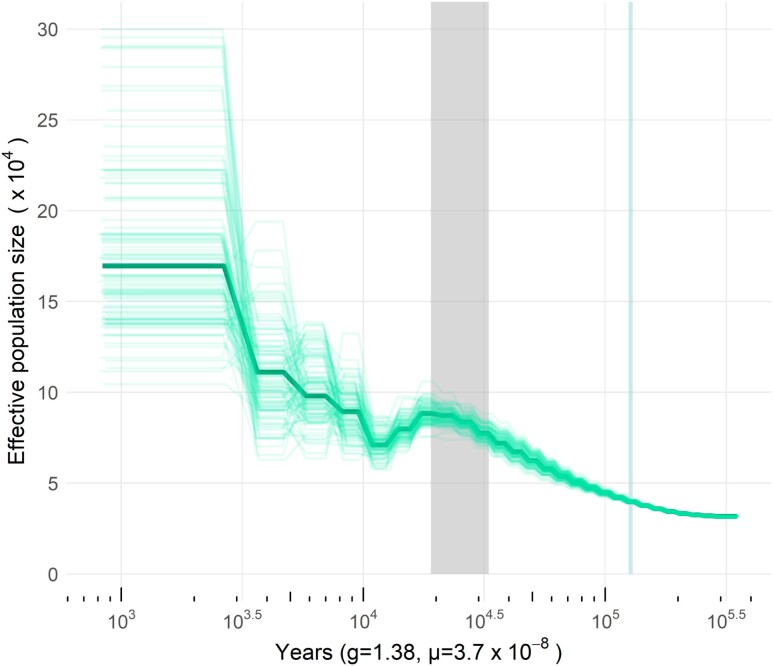
Historical effective population size of the pinfish as estimated by PSMC (Li and Durbin 2011). The bold green line shows the estimated effective population size over time from the full dataset, and the lighter green lines show results of 100 bootstrap replicates. The dark gray vertical bar highlights the timing of the last glacial maximum (LGM), and the light blue vertical bar shows the timing of the last interglacial stage (LIG).

The historical effective population size of the pinfish as inferred by PSMC is shown in Fig. 4. Scaling the results based on an estimated generation time of 1.38 years and a neutral mutation rate of 3.7*10^−8^ substitutions/site/generation revealed a gradual increase in *N_e_* from *ca.* 200 to 20 ka, with a peak *N_e_* of ∼90,000 occurring at 20 ka. This was then followed by a small decline in effective population size from 20 to 10 ka, and finally, a rapid increase from 10 ka until ∼1 ka, with *N_e_* reaching its maximum value of 175,000 ∼1,000 years ago. There is, however, a higher variance observed in the bootstrap replications from 20 ka to the present day (Fig. 4), suggesting that these estimates of recent *N_e_* should be interpreted with caution.

## Discussion

The pinfish genome assembly represents the first genome of a western Atlantic sparid and only the third chromosome-scale assembly for the family. Given the cultural, economic, and ecological significance of this group, this provides a valuable reference for future studies and sheds light on the evolutionary history of an understudied species. From this genome, we identified molecular signatures potentially associated with ontogenetic habitat shifts and dietary changes that characterize this species. We also used this resource to infer historical patterns of population expansion and contraction, which may be linked to ancient climatic oscillations.

### Genome assembly and annotation

The pinfish genome assembly is 785.3 Mb in length, and comprised of 24 scaffolded chromosomes, which is consistent with both the known karyotype of *L. rhomboides* (2*n* = 48, Fitzsimons and Parker 1985) and the approximate genome sizes of other fish in family Sparidae (Table 1). Our assembly shows similar contiguity and completeness to the assemblies of *S. aurata* and *A. latus* available in NCBI's RefSeq collection (Table 1). The number of protein-coding genes identified in the *L. rhomboides* genome (24,634) is also consistent with these RefSeq genomes (*S. aurata*: 25,735 coding genes; *A. latus*: 23,786 coding genes), providing further evidence that the pinfish genome is highly complete and may serve as a useful reference for future studies of sparid genomics.

### Genomic rearrangements over evolutionary time

The genome of the pinfish shows strongly conserved macrosynteny with that of the Japanese pufferfish (*Takifugu rubripes*), with only 2 putative instances of chromosomal fission or fusion having occurred (Supplementary Fig. 1). This conservation of large-scale synteny is particularly striking, given that pufferfishes are known to have undergone extensive reductions in genome size (Neafsey and Palumbi 2003). Our observation of extensive intrachromosomal rearrangement but few interchromosomal changes further corroborates the findings of Amores *et al*. (2014), who noted that teleost karyotypes appear to be surprisingly stable over deep evolutionary time.

In comparing the genome of the pinfish to those of *S. aurata* and *A. latus*, we found remarkable conservation of synteny across their 24 chromosomes (Figs. 2, Supplementary Figs. 2 and 4), particularly considering the time scale over which these three species have diverged. *L. rhomboides* diverged from *S. aurata* and *A. latus* ∼49 million years ago (Santini *et al*. 2014), which is comparable to the divergence timing between dogs (haploid *n* = 39) and cats (haploid *n* = 19; Kumar *et al*. 2017). This strong conservation of chromosome-scale synteny across family Sparidae is not unusual for teleost fishes but rather provides additional evidence in support of the long-term patterns of karyotype stability seen in this group (Amores *et al*. 2014).

Despite the broad conservation of chromosome number and structure, 2 large-scale intrachromosomal inversions were observed between the genome of the pinfish and those of *S. aurata* and *A. latus* (Figs. 2, Supplementary 2–5, Supplementary Tables 3 and 4). These inversions were common to both pairwise comparisons (*L. rhomboides* vs *S. aurata* and *L. rhomboides* vs *A. latus*), suggesting they are likely changes that occurred after the split between *L. rhomboides* and the lineage leading to *S. aurata* and *A. latus*. The 4.1 Mb inversion on *L. rhomboides* chromosome 13 contained several genes involved in nervous and sensory system development. It is possible that the genomic rearrangement of these sensory system genes could have played a role in the evolution of the ability of the pinfish to tolerate a wide array of habitats across its lifetime. Pinfish occupy shallow, nearshore seagrass beds during their larval and juvenile phases, which represent a starkly different habitat from the darker, deeper, and colder areas that they migrate to and inhabit as adults (Caldwell 1957; Darcy 1985). Many species of fish have evolved finely tuned sensory systems for life in a specific habitat (Ladich 2014; Musilova *et al*. 2019; Eaton *et al*. 2021), but when an organism's preferred habitat shifts over the course of an individual's lifetime, modifications to the sensory system often occur, including changes in sensory receptor expression (Cortesi *et al*. 2016). It is possible that genomic structural changes in the chromosome 13 inversion may have facilitated changes in sensory system gene expression that eventually played a role in the evolution of the ontogenetic habitat shift seen in the pinfish, particularly given that *A. latus* and *S. aurata* do not appear to undergo similar habitat shifts (Platell *et al*. 2007; Basurco *et al*. 2011). Based on the available data, it is not possible to determine whether this genomic rearrangement happened in the pinfish lineage or in the lineage containing the common ancestor to *S. aurata* and *A. latus*, but regardless, a major chromosomal inversion involving genes of the sensory system could have played a role in the diversification of these clades. Future studies are necessary to evaluate the precise functional role, if any, of the inversions detected in this study.

### Gene family expansions are implicated in the biology of the pinfish

In a comparative analysis of gene family evolution across the teleost tree of life, we found several gene families that were rapidly expanding or contracting in the terminal branch leading to the pinfish. Most notably, there was a significant expansion of digestion-associated genes, which could be associated with the evolution of dietary breadth in the pinfish. Specifically, we found evidence of multiple tandem duplications of *trypsin-like* genes (Fig. 3), resulting in a cluster of 11 *trypsin*-*like* genes within a 57-kb window on chromosome 5. *Trypsin*, a serine protease, plays a crucial role in the cleavage and digestion of dietary protein (Solovyev *et al*. 2023). Though the role and function of each of these *trypsin-like* genes in the pinfish has yet to be determined, it is possible that tandem duplication of *trypsin-like* genes and subsequent molecular evolution of different isoforms of this protease could improve the ability of the pinfish to digest a wider array of proteins. Given that most species in family Sparidae are carnivorous (Basurco *et al*. 2011), expansions of *trypsin-like* enzymes in the pinfish may have facilitated their adoption of an herbivorous lifestyle in adulthood. Similar expansions of *trypsin-like* serine proteases have been implicated in the adaptive evolution of generalist feeding habits in species of *Drosophila* (Li *et al*. 2012). With this in mind, future work should focus on understanding the potential role of these duplicated genes in digestion throughout the ontogeny of the pinfish.

In the lineage leading to family Sparidae as a whole, we found rapid contraction of several gene families involved in olfaction. This result was not surprising, because across the fish tree of life, there is often extreme variability in the olfactory receptor gene repertoire (Niimura 2009), even among species in the same family. Liu *et al*. (2021) inferred that virtually all marine fish lineages have undergone contractions in olfactory receptor number (as compared to the MRCA of all Osteichthyes), which is further corroborated by our findings.

### Historical population size corresponds to climatic shifts

The reconstruction of paleodemography showed evidence of effective population expansion and contraction consistent with the known timing of sea level fluctuations in the northern Gulf of Mexico (Fig. 4). Starting from 100 to 200 thousand years ago (ka) and continuing until 20 ka, there was a gradual, consistent rise in *L. rhomboides N_e_*. The beginning of this slow increase in effective population size roughly coincides with the timing of the last interglacial stage (LIG; *ca.* 125 ka), a geologic period during which sea surface temperatures were approximately 2°C warmer than preindustrial levels, and sea levels were 6–9 m higher than the present (Kopp *et al*. 2009; Donoghue 2011). During the LIG, much of south Florida was submerged in a shallow sea, and marine organisms could move more freely between the area now known as the Gulf of Mexico and the western Atlantic Ocean, potentially increasing their range and effective population size. Relatively slow cooling and gradual reductions in sea levels over the 100,000-year period from the LIG until ∼20 ka likely resulted in stable climatic conditions which could have favored additional population growth, culminating in the peak in *N_e_* observed around 20 ka (Fig. 4; Balsillie and Donoghue 2004; Donoghue 2011). Although our effective population size inference from 20 ka to present day is likely less accurate than our estimates across deeper time, as indicated by the increased variance in bootstrap estimates, we do see some interesting trends from 20 ka onwards which may be further correlated with ancient climatic changes. Notably, following the peak in effective population size which occurred around 20 ka, we see a gradual decline in effective population size, which also corresponds temporally with a major historical climatic event—the end of the Last Glacial Maximum (LGM). The reduction in effective population size at this time could be related to changes in habitat availability over a relatively short period of time, as previously shallow coastal regions saw rapid increases in-depth and ecological changes (Donoghue 2011). The final stabilization and eventual rapid growth in effective population size of the pinfish (occurring from *ca.* 10 ka—present) also corresponds temporally to the point at which the Laurentide Ice Sheet had fully disappeared, and the Gulf of Mexico attained present-day sea levels (∼8 ka; Dyke and Prest 1987; Donoghue 2011), which presented relatively stable conditions for population growth.

The co-occurrence of historical population expansions and contractions with ancient climatic changes in this species sheds further light on how pinfish may be affected by anthropogenic climate change. Previous work has shown that pinfish undergo a suite of detrimental molecular and physiological changes in response to acute warming (Eaton *et al*. 2022). This, in conjunction with our finding of historical population declines during a period of rapid climatic change and ocean warming (i.e. the end of the LGM), indicates that this ecologically crucial species may be more vulnerable to climatic warming than previously reported.

### The genome of the pinfish as a valuable tool for understanding coastal fish biology

The pinfish genome assembly represents an important step toward a better understanding of coastal and offshore ecosystem dynamics of the western Atlantic Ocean. Though pinfish are among the most abundant members of nearshore seagrass ecosystems in the Gulf of Mexico (Darcy 1985), they have remained understudied in terms of their functional genomics. This study represents the first step in bridging this knowledge gap, providing a high-quality genome for the pinfish, which can ultimately be used as a reference for studies of population genomics, local adaptation, and responses to environmental variation. Additionally, we have identified several unique genomic features that may underlie the distinct biology and ecology of the pinfish, including duplications of *trypsin-like* digestive enzymes and a chromosomal inversion enriched with sensory system-associated genes. Ultimately, this genome represents a valuable step forward in understanding the biology of a key species in subtropical and temperate coastal ecosystems of the western Atlantic.

## Supplementary Material

jkae096_Supplementary_Data

## Data Availability

All sequencing data have been deposited in GenBank under BioProject number PRJNA1039866. Specifically, the raw Nanopore and Illumina reads are accessible from GenBank under the BioSample numbers SAMN38231053 and SAMN38231054, respectively. The whole genome assembly has been deposited at GenBank under the accession JBBNBP000000000. The version described in this paper is version JBBNBP010000000. The complete assembly and all associated annotation files are also available on Figshare at the following: DOI: https://doi.org/10.25387/g3.24639582. Complete details of the bioinformatic pipeline followed to assemble and annotate the genome, as well as conduct the subsequent analyses, are described in the Supplemental Methods. Other scripts and bioinformatic details associated with this project are publicly available at https://github.com/kmeaton/Pinfish_Genome. Supplemental material available at G3 online.
