# Supplementary material for: The chromosome-scale reference genome for the pinfish (Lagodon rhomboides) provides insights into their evolutionary and demographic history
Source: G3 (Bethesda). 2024 May 13;14(7):jkae096. doi: 10.1093/g3journal/jkae096 (PMC11228864; doi:10.1093/g3journal/jkae096)
Supplement: jkae096_Supplementary_Data [file jkae096_supplementary_data.pdf]

<u>Supplemental Material for:</u>

The chromosome-scale reference genome for the pinfish (*Lagodon rhomboides*) provides insights into their evolutionary and demographic history

Katherine M. Eaton, Trevor J. Krabbenhoft, Nathan J.C. Backenstose, and Moisés A. Bernal

Supplemental Methods:

Description of long-read DNA extraction technique modifications

Long-read DNA was extracted using the Qiagen Genomic-tip 500/G kit, following the manufacturer's protocol, with slight modifications. Approximately 400 mg of gill or muscle tissue was finely ground in liquid nitrogen using a mortar and pestle. The homogenized tissue was added to 19 mL of buffer G2, 38 µL RNase A, and 1 mL Qiagen Protease, and incubated overnight at 50°C. Following overnight digestion, samples were purified using the genomic-tip column per the manufacturer's instructions, and long-read DNA was precipitated in 10.5 mL of cold isopropanol. DNA was spooled on a glass rod and dissolved in 1 mL of TE buffer. DNA was quality-checked visually on a 1% agarose gel and quantified using an Invitrogen Qubit 4 Fluorometer.

Assembly commands and parameters

Raw Illumina reads were trimmed to remove adapters and low-quality sequences using the program cutadapt v 2.3 (Martin 2011), with the following parameters: -a AATGATACGGCGACCACCGAGATCTACACTCTTTCCCTACACGACGCTCTTCCGATCT -A GATCGGAAGAGCACACGTCTGAACTCCAGTCACATCACGATCTCGTATGCCGTCTTCTGCTTG --trim-n -q 10 --cores=20.

Raw nanopore reads were assembled using Flye v. 2.8-b1674 (Kolmogorov et al. 2019), with the following parameters: --nano-raw -g 1g --iterations 3. The trimmed Illumina reads were then used to polish this initial genome assembly. Briefly, reads were mapped to the preliminary assembly using the program bwa mem v. 0.7.12-r1039 (Li 2013), with default parameters. Output SAM files were converted to BAM format, sorted, and indexed using samtools v. 1.9 (Li et al. 2009), with the following commands: samtools view -@ 40 -S -q 15 -b | samtools sort -@ 40

sorted.bam; samtools index sorted.bam. Pilon v. 1.23 (Walker et al. 2014) was then used to polish the assembly, using the BAM alignments and default parameters.

Description of Kraken database to detect microbial and non-target reads

To remove any unscaffolded contigs that were the result of microbial or non-target reads, we created a custom taxonomic sequence classification database with the program Kraken 2 v. 2.1.2 (Wood et al. 2019). For this, the taxonomic information and genome sequences from the viral, bacterial, and archaeal domains, as well as the complete human genome sequence and the collection of plasmid sequences available on NCBI's RefSeq database were downloaded. Additionally, we added the complete genome sequences and taxonomic information for three fish species with publicly available genomes on NCBI: *Danio rerio* (BioProject #PRJNA13922), *Larimichthys crocea* (BioProject #PRJNA354443), and *Lutjanus erythropterus* (BioProject #PRJNA662638). The complete database was built using the kraken2-build function in Kraken 2, and then contigs in the pinfish genome were searched and taxonomically classified based on this database, using Kraken 2 with the default parameters. Contigs classified as non-fish in origin were removed from the assembly using the program SeqKit v. 0.14.0 (Shen et al. 2016). The resulting scaffolds were named and ordered according to length. The final assembly was assessed for completeness using BUSCO v. 5.1.2 (Manni et al. 2021), searching against the *actinopterygii_odb10* database, using the gene predictor MetaEuk (Levy Karin et al. 2020).

Annotation procedure and parameters

We used RepeatModeler v. 2.0.1 (Flynn et al. 2020) to characterize transposable element families in our genome. Repeat families identified from RepeatModeler were used as a custom repeat library for repeat masking by RepeatMasker v. 4.1.1 (Smit et al. 2013). We then performed two rounds of genome annotation using MAKER v. 3.01.03 (Cantarel et al. 2008), following the

pipeline provided by Card et al. (2019). We supplied repeat evidence from RepeatModeler, protein evidence from the closely related gilthead seabream (*Sparus aurata*) and the zebrafish (*Danio rerio*), obtained from Ensembl (Howe et al. 2021, www.ensembl.org, Ensembl release 103), and expressed sequence tag (EST) evidence using the transcriptome assembly for *L. rhomboides* (Eaton et al. 2022). Genes were initially predicted using Augustus (Stanke et al. 2006), based on gene models from *D. rerio*, in addition to being inferred directly from the provided protein and EST datasets. Prior to the second round of annotation, we generated species-specific hidden Markov models (HMMs) for the gene predictors SNAP v. 2006-07-28 (Korf 2004) and Augustus (Stanke et al. 2006) using predicted genes from round 1 of MAKER. These pinfish-specific HMMs for SNAP and Augustus were used, along with the predicted ESTs, proteins, and repeats from round 1 of MAKER, to perform the second round of genome annotation using the MAKER pipeline. Further annotation efforts past this second round of MAKER did not result in improvement to our gene predictions. To validate our results, we used InterProScan v. 5.52-86.0 (Jones et al. 2014) to identify proteins in our annotation that contained at least one recognizable protein domain found in the Pfam database of protein families, specifying the following options: -cpu 10 -appl Pfam -iprlookup -goterms -f tsv.

Predicted proteins from the MAKER output were annotated in a series of iterative BLAST searches for sequence similarity to proteins from publicly available fish genomes. Using the program BLAST+ v. 2.13.0 (Altschul et al. 1990, Camacho et al. 2008), we searched the predicted proteins from the *L. rhomboides* genome against a database of peptide sequences from the *S. aurata* genome downloaded from Ensembl (Cunningham et al. 2022, Ensembl release 107), using the command 'blastp' and specifying an e-value cutoff of $1e^{-10}$. BLAST matches were filtered to only include high-quality matches (i.e., matches with query

coverage > 50% and percent identity ≥ 75%) and the best match for each protein was identified based on the highest bit-score, using a custom bash script. This process was then repeated for predicted proteins in the pinfish genome that did not have a high-quality match in the *S. aurata* database, using the same search parameters and searching against peptides from the large yellow croaker genome (*Larimichthys crocea*) and then the zebrafish genome (*Danio rerio*), downloaded from Ensembl (Cunningham et al. 2022, Ensembl release 107).

Synteny analyses

To examine genomic structure and large-scale chromosomal rearrangements over evolutionary time, we used the program SynMap2 (Haug-Baltzell et al. 2017) with default parameters implemented in CoGe (Lyons and Freeling 2008) to compare the structure of the pinfish genome to that of the gilthead seabream (*Sparus aurata*; NCBI assembly accession number: GCA_900880675.1), and the yellowfin seabream (*Acanthopagrus latus*; NCBI assembly accession number: GCF_904848185.1), and the Japanese puffer (*Takifugu rubripes*; NCBI assembly accession number: GCF:_901000725.2). To aid in visualization and interpretation of the synteny results, we also generated synteny plots using the program D-GENIES (Cabanettes and Klopp 2018), which generates dot plots of genomic alignments created via the program Minimap v. 2.26 (Li 2018). Syntenic matches were plotted using Circos v. 0.69-9 (Krzywinski et al. 2009). Large-scale inversions (>4 Mb in length) were identified by eye on the resulting plots, and then filtered to exclude those which may be the results of scaffolding errors. Briefly, we determined the genomic coordinates of the inversion breakpoints and compared them with our scaffolding data. For those inversions on which one or more breakpoints coincided with a gap between scaffolded contigs, we assumed the possibility of a scaffolding error and removed that inversion from

downstream analyses. This approach, while conservative, minimizes the possibility of a Type I error in inversion detection. The genomic content of putative inversions was then investigated based on the annotation generated from MAKER. We used Fisher's exact test to determine if certain Gene Ontology (GO) terms were overrepresented among the set of genes found in each inversion, as compared to the rest of the genome.

Gene family evolution

To gain insight into the functional genomics and evolutionary history of the pinfish, we assessed phylogenetic patterns of gene family expansion and contraction using CAFE v. 5.0 (Mendes et al. 2020). This program uses a birth/death process to model gains and losses of genes in particular gene families across a time-calibrated phylogenetic tree, inferring the most likely number of genes in each gene family at every internal node of the phylogeny. It then estimates a maximum-likelihood global evolutionary rate for all gene families across the tree. Then, for each gene family, every branch of the phylogeny is assessed to determine if the rate of gene gain or loss is significantly greater than the global rate, which indicates a significantly rapidly evolving gene family at that point in time. We downloaded peptide sequences from 18 publicly available teleost fish genomes (Table S1). For each species, we identified the longest isoform of each gene using the python script "longest_iso.py" provided by the CAFE developers, and then further filtered to remove duplicate sequences with SeqKit v. 0.14.0 (Shen et al. 2016). To identify families of orthologous genes and to build a phylogeny for use in CAFE, we used the program OrthoFinder v. 2.4.0 with default parameters (Emms and Kelly 2019). The output table containing the gene counts per orthogroup for each species was filtered to remove gene families with large copy number variance from the dataset, and then the filtered table was used as input for CAFE.

To generate a time-calibrated phylogenetic tree for use in CAFE, we first identified single-copy orthologs that were present in all 19 species, using the output of OrthoFinder. For each single-copy orthogroup, we ran the custom python script "extract_cds.py" (modified from Jessie Pelosi and Kasey K. Pham) to extract coding sequences for single-copy orthologs. To ensure that we had isolated the CDS associated with the longest isoform of each gene, we used a custom bash script and the program SeqKit (v. 0.14.0, Shen et al. 2016). We then aligned the coding sequences for each single-copy orthogroup using the program MACSE v. 2.06 (Ranwez et al. 2018) and constructed a maximum-likelihood phylogenetic tree using IQ-TREE v. 1.6.12 using ModelFinder for extended model selection (Nguyen et al. 2015, Kalyaanamoorthy et al. 2017, Hoang et al. 2018) with the following parameters: -m MFP -alrt 1000 -bb 1000 -nt 2. The individual gene trees for each orthogroup were then used to infer a species tree using the program ASTRAL v. 5.7.8 (C. Zhang et al. 2018). We then generated a final maximum-likelihood tree from a concatenated alignment of all single-copy orthologs using IQ-TREE as above (parameters: -g ASTRAL_guide_tree.tre -alrt 1000 -bb 1000 -spp partition.nex -nt 10), partitioning by locus and using the previously identified best-fit substitution model for each ortholog (Chernomor et al. 2016), and constraining the topology of the tree to match that of the ASTRAL-generated species tree. This final maximum-likelihood tree was then time-calibrated using the program treePL (Smith and O'Meara 2012), using seven fossil calibrations (Table S2).

Using the table of gene counts per orthogroup (or gene family) as identified by OrthoFinder and the ultrametric phylogeny, we ran CAFE v. 5.0 (Mendes et al. 2020) to identify gene families that have undergone significantly rapid expansions or contractions in gene copy number over evolutionary time. An initial run of CAFE estimated a model accounting for genome assembly error across our 19 taxa (specifying the parameter -e), which was then used in a second round of

CAFE, to determine the global gene birth-death rate (λ) with two discrete evolutionary rate categories (-k 2). The output from CAFE was filtered manually to identify gene families that were significantly rapidly evolving ($p < 0.05$). To further investigate the functional significance of these families, we identified Gene Ontology (GO) categories that were overrepresented among the significantly rapidly evolving gene families using Fisher's exact test, based on the bioinformatic pipeline and code provided by Wright et al. (2015).

Demographic history analyses

To estimate patterns of historical population demography in the pinfish, we used the program PSMC (Li and Durbin 2011). PSMC calculates historical recombination events and the most recent common ancestor of alleles at a given locus across a single diploid genome to infer the coalescent rate within a time period, which has an inverse relationship to effective population size (Li and Durbin 2011). Briefly, we mapped the trimmed and cleaned Illumina reads back to the final genome assembly using the program bwa-mem v. 0.7.17-r1188 (Li 2013), and then sorted and indexed the aligned reads using the program samtools v. 1.7 (Li et al. 2009). We called SNPs for this alignment using the program bcftools v. 1.7 (Li 2011), using the command "mpileup" with the flag "-C 50", followed by the command "call" using the "-c" option to specify the consensus calling model. The resulting output VCF file was then converted into FASTQ format using the command "vcfutils.pl vcf2fq" from the program bcftools (Li 2011), specifying a minimum read depth of 10 and a maximum read depth of 120. The resulting FASTQ file was then used as input to run PSMC (Li and Durbin 2011) v. 0.6.5-r67, thereby estimating historical effective population size. We first did an initial run of the software, specifying -N25 (setting the maximum number of iterations to 25), -t5 (setting the maximum 2N0 coalescent time to 5), -r5 (setting the initial theta/rho ratio to 5), and -p "1*4+25*2+1*4+1*6" (setting the atomic time intervals), based on

parameters previously applied to similar analyses in threespine stickleback (Liu et al. 2016, Kirch et al. 2021). We then performed 100 bootstrap replicate runs, using the same parameters as above. The results were scaled to real time and plotted, assuming a generation time of 1.38 years, based on von Bertalanffy growth parameters for *L. rhomboides* estimated by Nelson (2002), and a mutation rate of $3.7*10^{-8}$ substitutions/nucleotide/generation (as in Kirch et al. 2021).

Supplementary Tables.

Table S1. Publicly available teleost fish genomes used in the gene family evolution analysis.

Species	Assembly accession number	Reference
Acanthochromis polyacanthus	GCA_002109545.1	Ensembl release 107 (Cunningham et al. 2022)
Amphiprion ocellaris	GCA_002776465.1	Ensembl release 107 (Cunningham et al. 2022)
Danio rerio	GCA_000002035.4	Ensembl release 107 (Cunningham et al. 2022)
Dicentrarchus labrax	GCA_905237075.1	Ensembl release 107 (Cunningham et al. 2022)
Gadus morhua	GCA_902167405.1	Ensembl release 107 (Cunningham et al. 2022)
Haplochromis burtoni	GCA_000239415.1	Ensembl release 107 (Cunningham et al. 2022)
Ictalurus punctatus	GCA_001660625.1	Ensembl release 107 (Cunningham et al. 2022)
Labrus bergylta	GCA_900080235.1	Ensembl release 107 (Cunningham et al. 2022)
Lagodon rhomboides	PRJNA1039866	This study
Larimichthys crocea	GCA_000972845.2	Ensembl release 107 (Cunningham et al. 2022)
Lates calcarifer	GCA_900066035.1	Ensembl release 107 (Cunningham et al. 2022)
Lepisosteus oculatus	GCA_000242695.1	Ensembl release 107 (Cunningham et al. 2022)
Mola mola	GCA_001698575.1<br>Annotation available at: http://dx.doi.org/10.5524/100214	Pan et al. 2016
Oreochromis niloticus	GCA_001858045.3	Ensembl release 107 (Cunningham et al. 2022)
Pagrus major	Raw reads: NCBI BioProject PRJNA480768<br>Assembly and annotation available at: https://doi.org/10.6084/m9.figshare.6962867.v1	Shin et al. 2018
Poecilia formosa	GCA_000485575.1	Ensembl release 107 (Cunningham et al. 2022)
Sander lucioperca	GCA_008315115.1	Ensembl release 107 (Cunningham et al. 2022)
Seriola dumerili	GCA_002260705.1	Ensembl release 107 (Cunningham et al. 2022)
Tetraodon nigroviridis	TETRAODON 8.0	Ensembl release 107 (Cunningham et al. 2022)

Table S2. Calibration points for nodes in the ultrametric phylogeny generated for the analysis of gene family evolution.

Clade	Fossil	Minimum age	Maximum age	Node calibrated	Reference
Crown Holostei	*Watsonulus eugnathoides*	250	--	MRCA *Lepisosteus oculatus – Danio rerio*	Benton et al. 2015
Crown Euteleostei	*Leptolepides haerteisi*	150.94	242	MRCA *Danio rerio – Gadus morhua*	Benton et al. 2015
Crown Acanthomorpha	*Stichocentrus liratus*	98	158.3	MRCA *Gadus morhua – Poecilia formosa*	Benton et al. 2015
Crown Atherinomorpha	*Ramphexocetus volans*	49.11	130.8	MRCA *Poecilia formosa – Oreochromis niloticus*	Benton et al. 2015
Stem Tetraodontoidei	*Balkaria histiopterygia*	55.8	--	MRCA *Tetraodon nigroviridis – Mola mola*	Bannikov et al. 2017
Crown Sparidae	*Sciaenurus bowerbanki*	52	--	MRCA *Pagrus major – Lagodon rhomboides*	Santini et al. 2014
Stem Pomacentridae	*Palaeopomacentrus orphae*	50	--	MRCA *Acanthochromis polyacanthus – Oreochromis niloticus*	Bellwood and Sorbini 1996

Table S3. Genomic coordinates of large-scale inversions when comparing the *L. rhomboides* and *S. aurata* genomes.

L. rhomboides chromosome	*L. rhomboides* start (bp)	*L. rhomboides* end (bp)	*S. aurata* chromosome	*S. aurata* start (bp)	*S. aurata* end (bp)	Inversion length in *L. rhomboides* genome (Mbp)
Lrho13	11,402,433	15,540,636	Saur15	11,500,862	15,764,842	4.14
Lrho23	230,348	5,524,276	Saur10	29,939,147	35,423,347	5.29

Table S4. Genomic coordinates of large-scale inversions when comparing the *L. rhomboides* and *A. latus* genomes.

L. rhomboides chromosome	*L. rhomboides* start (bp)	*L. rhomboides* end (bp)	*A. latus* chromosome	*A. latus* start (bp)	*A. latus* end (bp)	Inversion length in *L. rhomboides* genome (Mbp)
Lrho13	11,453,722	15,540,636	Alat15	10,264,622	14,204,987	4.09
Lrho23	249,375	5,491,795	Alat10	17,826,383	22,344,748	5.24

Table S5. Significantly enriched Gene Ontology (GO) terms in large-scale inversions between the *L. rhomboides* and *S. aurata* genomes.

**Inversion location (*L. rhomboides* genome)**	**GO term ID**	**GO term name**	**GO term category**	**Adjusted p-value**
Lrho13	GO:0060122	Inner ear receptor cell stereocilium organization	Biological process	0.00301085
Lrho13	GO:0050910	Detection of mechanical stimulus involved in sensory perception of sound	Biological process	0.00421162
Lrho13	GO:0050957	Equilibrioception	Biological process	0.00421162
Lrho13	GO:0060117	Auditory receptor cell development	Biological process	0.00421162
Lrho13	GO:0061512	Protein localization to cilium	Biological process	0.00421162
Lrho13	GO:0048667	Cell morphogenesis involved in neuron differentiation	Biological process	0.0052407
Lrho13	GO:0009612	Response to mechanical stimulus	Biological process	0.00700194
Lrho13	GO:0050974; GO:0050982	Detection of mechanical stimulus	Biological process	0.03068545
Lrho13	GO:0001755	Neural crest cell migration	Biological process	0.03383291
Lrho13	GO:0009913; GO:0035315	Epidermal cell differentiation	Biological process	0.03383291
Lrho13	GO:0031175	Neuron projection development	Biological process	0.03383291
Lrho13	GO:0060119	Inner ear receptor cell development	Biological process	0.03383291
Lrho13	GO:0006897	Endocytosis	Biological process	0.04147989
Lrho13	GO:0098858	Actin-based cell projection	Cellular component	0.0079
Lrho13	GO:0043005	Neuron projection	Cellular component	0.011
Lrho13	GO:0005929	Cilium	Cellular component	0.036

Supplemental Figures.

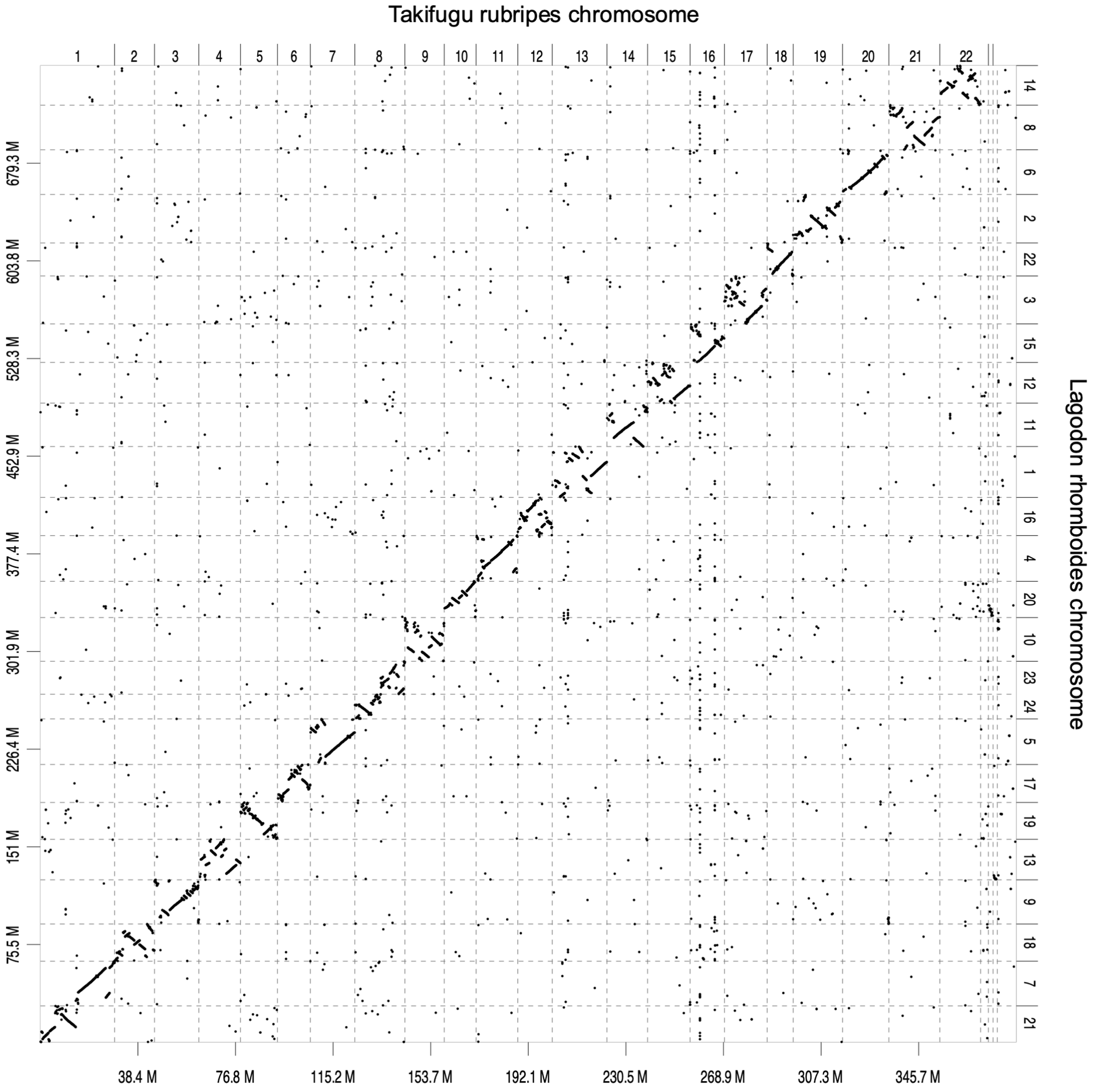

Figure S1. Synteny plot comparing the structure of the pinfish genome (y-axis) to the genome of the Japanese pufferfish (*Takifugu rubripes*, x-axis). Black dots represent syntenic regions (based on sequence similarity) shared between the two genomes.

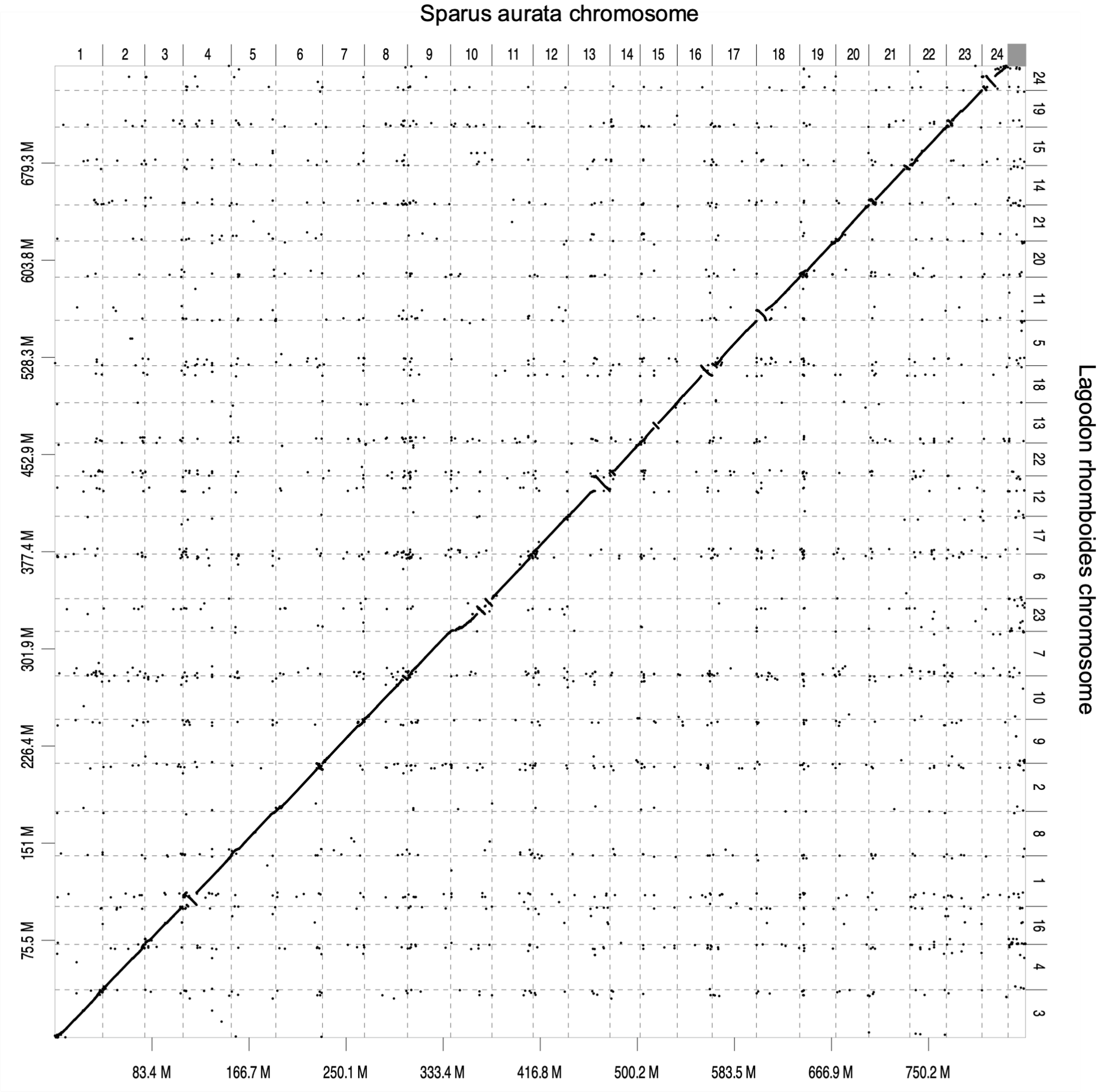

Figure S2. Synteny plot comparing structure of the pinfish genome (y-axis) to the genome of the gilthead seabream (*Sparus aurata*, x-axis). Black dots represent syntenic regions (based on sequence similarity) shared between the two genomes. Note the strong 1:1 relationship between syntenic chromosomes of each genome (i.e., the diagonal line in the center of the plot).

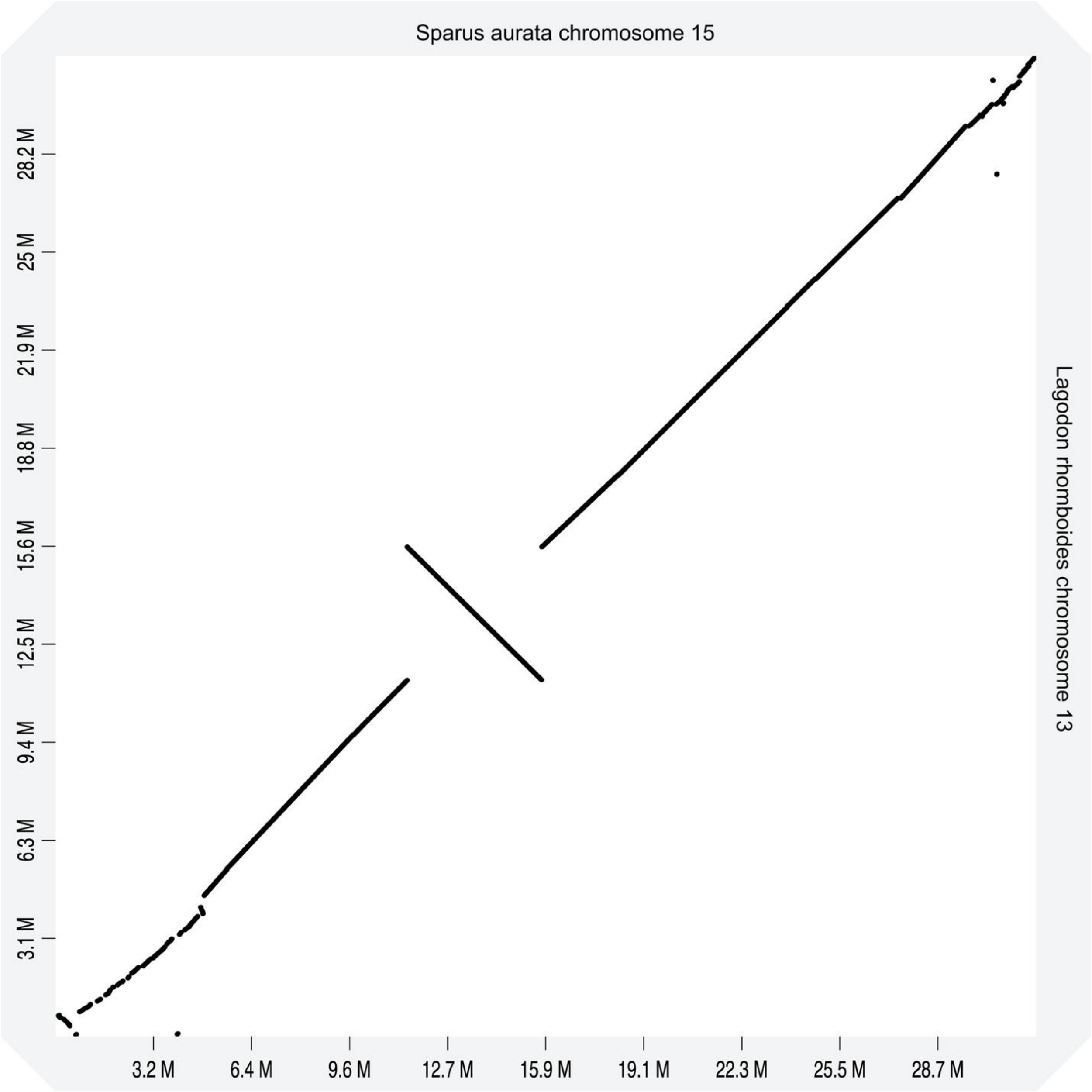

Figure S3. Close up plot showing the syntenic relationship between *L. rhomboides* chromosome 13 and *S. aurata* chromosome 15. Note the large chromosomal inversion that has occurred in the middle of pinfish chromosome 13.

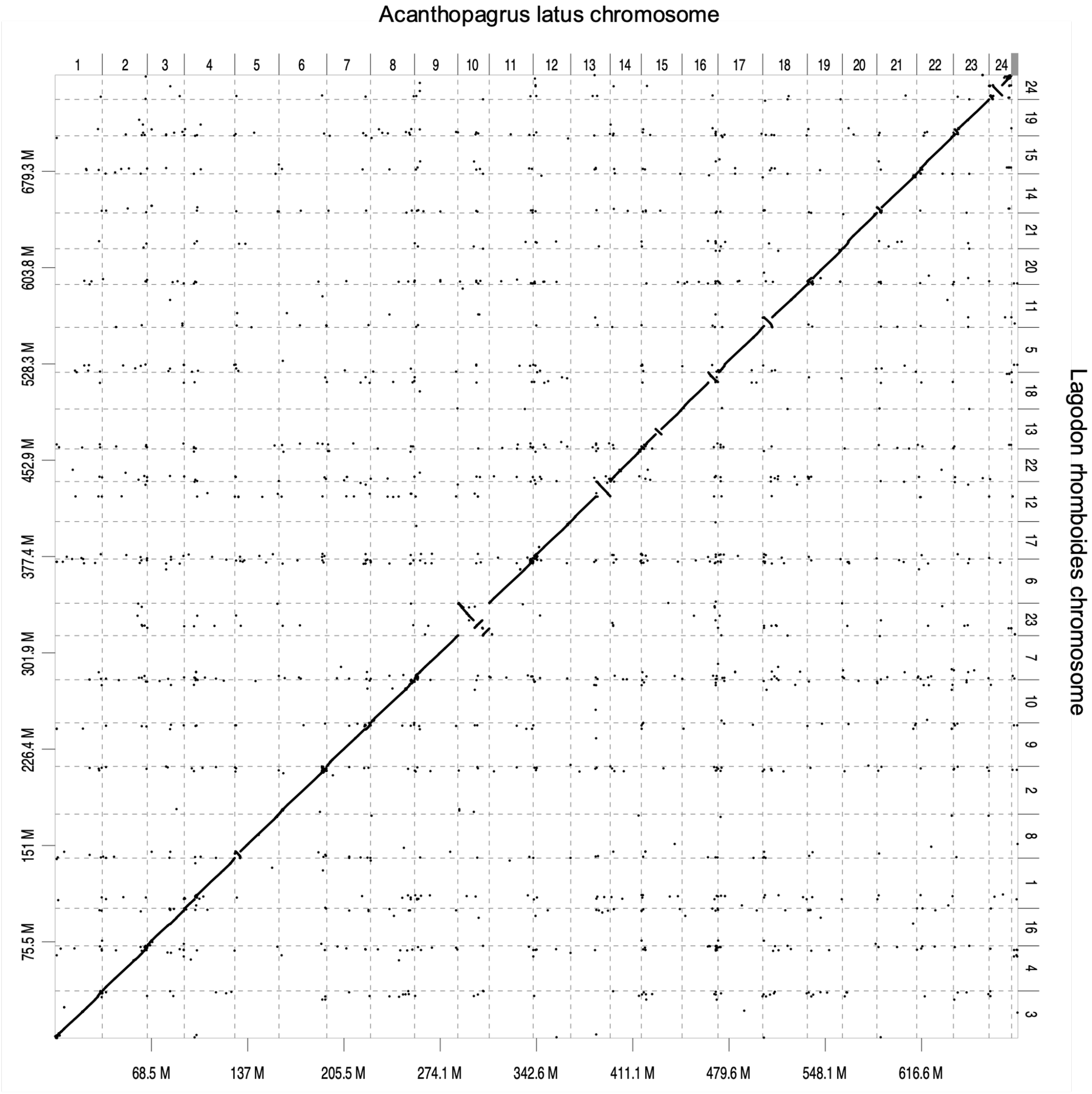

Figure S4. Synteny plot comparing structure of the pinfish genome (y-axis) to the genome of the yellowfin seabream (*Acanthopagrus latus*, x-axis). Black dots represent syntenic regions (based on sequence similarity) shared between the two genomes. Note the strong 1:1 relationship between syntenic chromosomes of each genome (i.e., the diagonal line in the center of the plot)..

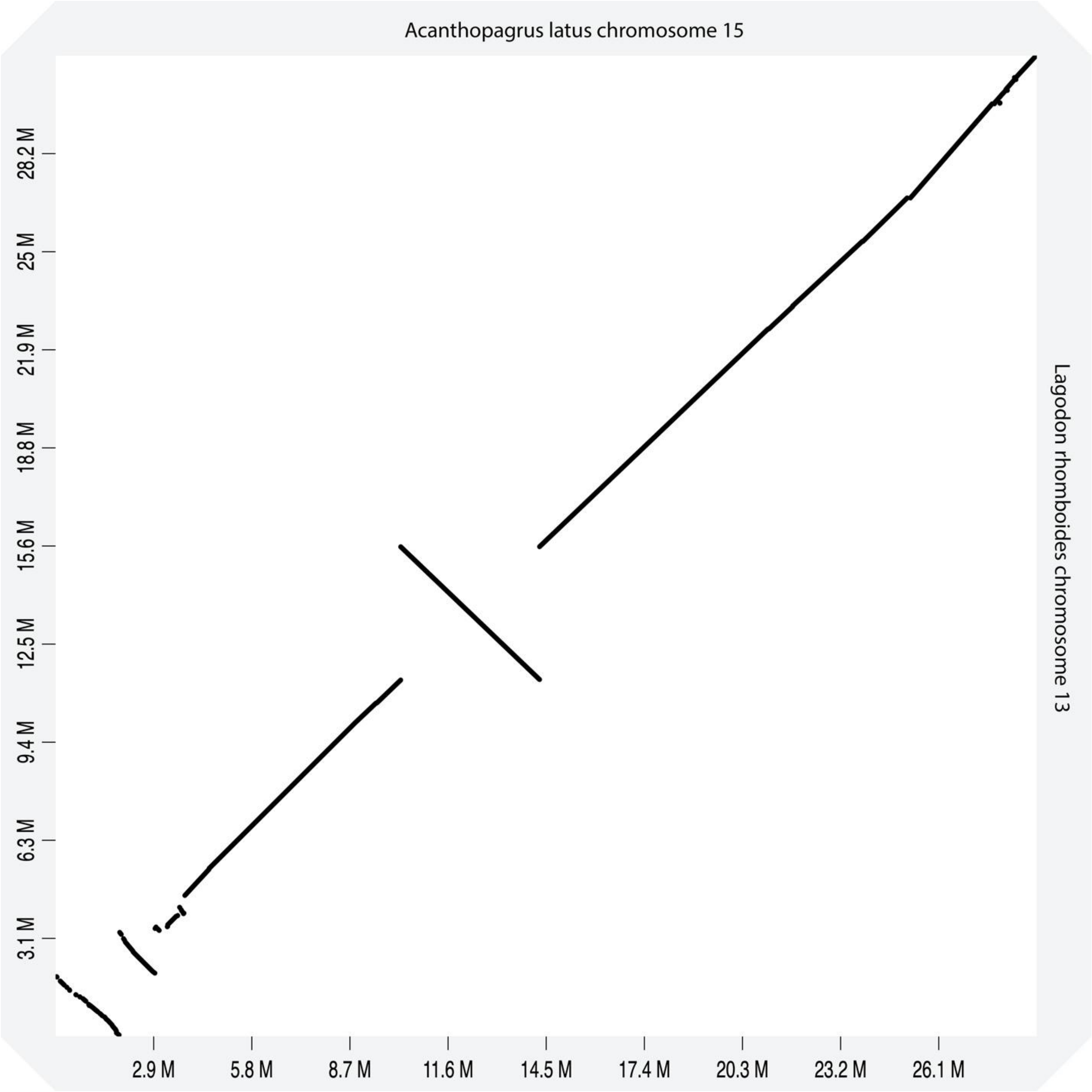

<u>Figure S5.</u> Close up plot showing the syntenic relationship between *L. rhomboides* chromosome 13 and *A. latus* chromosome 15. Note the large chromosomal inversion that has occurred in the middle of pinfish chromosome 13.

References

Altschul SF, Gish W, Miller W, Myers EW, Lipman DJ. 1990. Basic local alignment search tool. *J. Mol. Biol.* 215:403-410.

Bannikov AF, Tyler JC, Arcila D, Carnevale G. 2017. A new family of gymnodont fish (Tetraodontiformes) from the earliest Eocene of the Peri-Tethys (Kabardino-Balkaria, northern Caucasus, Russia). *Journal of Systematic Palaeontology*. 15(2):129-146.

Bellwood DR, Sorbini L. 1996. A review of the fossil record of the Pomacentridae (Teleostei: Labroidei) with a description of a new genus and species from the Eocene of Monte Bolca, Italy. *Zoological Journal of the Linnean Society*. 117(2):159-174.

Benton MJ, et al. 2015. Constraints on the timescale of animal evolutionary history. *Palaeontologia Electronica*. 18.1.1FC. 1-106.

Cabanettes F, Klopp C. 2018. D-GENIES: dot plot large genomes in an interactive, efficient and simple way. *PeerJ*. 6:e4958. https://doi.org/10.7717/peerj.4958.

Camacho C, et al. 2008. BLAST+: architecture and applications. *BMC Bioinformatics*. 10:421.

Cantarel BL, et al. 2008. MAKER: an easy-to-use annotation pipeline designed for emerging model organism genomes. *Genome Research.* 18:188-196.

Card DC, et al. 2019. Genomic basis of convergent island phenotypes in Boa constrictors. *Genome Biology and Evolution*. 11:3123-3143.

Chernomor O, von Haeseler A, Minh BQ. 2016. Terrace aware data structure for phylogenomic inference from supermatrices. *Systematic Biology*. 65(6):997-1008.

Cunningham F, et al. 2022. Ensembl 2022. *Nucleic Acids Res.* 50(1): D988-D995.

Eaton KM, Hallaj A, Stoeckel JA, Bernal MA. 2022. Ocean warming leads to increases in aerobic demand and changes to gene expression in the pinfish (*Lagodon rhomboides*). *Frontiers in Ecology and Evolution*. 9:809375.

Emms DM, Kelly S. 2019. OrthoFinder: phylogenetic orthology inference for comparative genomics. *Genome Biology*. 20:238. https://doi.org/10.1186/s13059-019-1832-y

Flynn JM, et al. 2020. RepeatModeler2 for automated genomic discovery of transposable element families. *PNAS*. 117:9451-9457.

Haug-Baltzell A, Stephens SA, Davey S, Scheidegger CE, Lyons E. 2017. SynMap2 and SynMap3D: web-based whole genome synteny browsers. Bioinformatics. 33(14):2197-2198.

Hoang DT, Chernomor O, von Haeseler A, Minh BQ, Vinh LS. 2018. UFBoot2: improving the ultrafast bootstrap approximation. Mol Biol. Evol. 35:518-522.

Howe KL, et al. 2021. Ensembl 2021. *Nucleic Acids Research.* 49:884-891.

Jones P, et al. 2014. InterProScan 5: genome-scale protein function classification. *Bioinformatics*. 30(9):1236-1240.

Kalyaanamoorthy S, Minh BQ, Wong TKF, von Haeseler A, Jermiin LS. 2017. ModelFinder: fast model selection for accurate phylogenetic estimates. *Nature Methods*. 14:587-589.

Kirch M, Romundset A, Gilbert MJP, Jones FC, Foote AD. 2021. Ancient and modern stickleback genomes reveal the demographic constraints on adaptation. *Current Biology*. 31(9):2027-2036.e8.

Kolmogorov M, Yuan J, Lin Y, Pevzner P. 2019. Assembly of long error-prone reads using repeat graphs. *Nature Biotechnology*. 37:540-546.

Korf I. 2004. Gene finding in novel genomes. *BMC Bioinformatics*. 5:59. https://doi.org/10.1186/1471-2105-5-59

Krzywinski MI, et al. 2009. Circos: an information aesthetic for comparative genomics. *Genome Res.* 19:1639-1645.

Levy Karin E, Mirdita M, Söding J. 2020. MetaEuk – sensitive, high-throughput gene discovery, and annotation for large-scale eukaryotic metagenomics. *Microbiome*. 8:48.

Li H, et al. 2009. The sequence alignment/map format and SAMtools. *Bioinformatics*. 25:2078-2079.

Li H, Durbin R. 2011. Inference of human population history from individual whole-genome sequences. *Nature*. 475:493-496.

Li H. 2011. A statistical framework for SNP calling, mutation discovery, association mapping, and population genetical parameter estimation from sequencing data. *Bioinformatics*. 27(21):2987-2993.

Li H. 2013. Aligning sequence reads, clone sequences and assembly contigs with BWA-MEM. *arXiv preprint*. arXiv:1303.3997.

Li H. 2018. Minimap2: pairwise alignment for nucleotide sequences. *Bioinformatics*. 34(18):3094-3100.

Liu S, Hansen MM, Jacobsen MW. 2016. Region-wide and ecotype-specific differences in demographic histories of threespine stickleback populations, estimated from whole genome sequences. *Molecular Ecology*. 25:5187-5202.

Lyons E, Freeling M. 2008. How to usefully compare homologous plant genes and chromosomes as DNA sequences. *The Plant Journal*. 53(4):661-673.

Manni J, Berkeley MR, Seppey M, Simão FA, Zdobnov EM. 2021. BUSCO update: novel and streamlined workflows along with broader and deeper phylogenetic coverage for scoring of eukaryotic, prokaryotic, and viral genomes. *Molecular Biology and Evolution*. 38:4647-4654.

Martin M. 2011. Cutadapt removes adapter sequences from high-throughput sequencing reads. *EMBnet.journal*. 17:10-12.

Mendes FK, Vanderpool D, Fulton B, Hahn MW. 2020. CAFE 5 models variation in evolutionary rates among gene families. *Bioinformatics*. 36:5516-5518.

Nelson GA. 2002. Age, growth, mortality, and distribution of pinfish (*Lagodon rhomboides*) in Tampa Bay and adjacent Gulf of Mexico waters. *Fish. Bull.* 100:582-592.

Nguyen L-T, Schmidt HA, von Haeseler A, Minh BQ. 2015. IQ-TREE: a fast and effective stochastic algorithm for estimating maximum likelihood phylogenies. *Mol. Biol. Evol.* 32:268-274.

Pan H, et al. 2016. The genome of the largest bony fish, ocean sunfish (*Mola mola*), provides insight into its fast growth rate. *GigaScience*. 5:s13742-016-0144-3.

Ranwez V, Douzery EJP, Cambon C, Chantret N, Delsuc F. 2018. MACSE v2: toolkit for the alignment of coding sequences accounting for frameshifts and stop codons. *Mol. Biol. Evol*. 35(10):2582-2584.

Santini F, Carnevale G, Sorenson L. 2014. First multi-locus timetree of seabreams and porgies (Percomorpha: Sparidae). *Italian Journal of Zoology*. 81(1):55-71.

Shen W, Le S, Li Y, Hu F. 2016. SeqKit: a cross-platform and ultrafast toolkit for FASTA/Q file manipulation. *PLoS One.* 11(10): e0163962.

Shin G-H, et al. 2018. First draft genome for red sea bream of family Sparidae. *Front. Genet.* 9:643. https://doi.org/10.3389/fgene.2018.00643

Smit AFA, Hubley R, Green P. 2013-2015. *RepeatMasker Open-4.0*. http://www.repeatmasker.org.

Smith SA, O'Meara BC. 2012. treePL: divergence time estimation using penalized likelihood for large phylogenies. *Bioinformatics*. 28(20):2689-2690.

Stanke M, Schöffmann O, Morgenstern B, Waack S. 2006. Gene prediction in eukaryotes with a generalized hidden Markov model that uses hints from external sources. *BMC Bioinformatics*. 7:62. https://doi.org/10.1186/1471-2105-7-62

Walker BJ, et al. 2014. Pilon: an integrated tool for comprehensive microbial variant detection and genome assembly improvement. *PLoS One*. 9(11):e112963.

Wood DE, Lu J, Langmead B. 2019. Improved metagenomic analysis with Kraken 2. *Genome Biology*. 20:257.

Wright RM, Aglyamova GV, Meyer E, Matz MV. 2015. Gene expression associated with white syndromes in a reef-building coral, *Acropora hyacinthus*. *BMC Genomics*. 16:371.

Zhang C, Rabiee M, Sayyari E, Mirarab S. 2018. ASTRAL-III: polynomial time species tree reconstruction from partially resolved gene trees. *BMC Bioinformatics*. 19:153. https://doi.org/10.1186/s12859-018-2129-y
